# Cancer and Ulceration of Colon

**Published:** 1831-01-01

**Authors:** 


### XVIII.

### HOTEL DIEU.

### Cancer and Ulceration of the Lower Portion of the Colon.

*Case.* A man, 31 years of age, of delicate constitution, had enjoyed good health till within a year of admission into the hospital. He then began to experience various painful sensations in different parts of the abdomen, but especially about the hollow of the left ilium. His appetite was not impaired, but his digestion was deranged, and he had frequent attacks of colic. The motions were generally liquid, consisting of bile or mucus—sometimes containing clots of blood. He soon began to lose both strength and flesh, and then a tumour appeared on the left flank, which was both visible and tangible. It was not till after eight months of suffering that this man applied at the Hôtel Dieu for relief. He was then in a state of extreme emaciation and debility—had lost his appetite—and was harrassed with diarrhœa. The suprà-iliac tumour was hard, and about the size of a man's fist. It was very tender to the touch. It was pronounced to be cancerous—and little doubt was entertained that it was in a state of ulceration. Nevertheless it was determined to employ *pressure* on the plan of Mr. Young in this country, and some ingenious instruments were contrived for effecting this measure. In six or eight days it is asserted that the volume of the tumour was considerably diminished, without any augmentation of the patient's sufferings. The pressure was continued for 15 days, when the tumour ceased to diminish, and became harder, as well as more tender. The diarrhœa and emaciation now made rapid progress—fever came on—the evacuations became extremely fetid, bloody, and puriform. Death soon put an end to the patient's sufferings.

*On dissection*, the stomach was found to be very large, softened, and reddened on its internal surface. The mucous membrane of the small and great intestines was developed in a most extraordinary manner, and of vast thickness. As the lower portion of the colon was approached, this thickening became more remarkable, and changed in fact into a sub-scirrhous condition. The sigmoid flexure of the colon was the seat of the tumour, which, indeed was formed by the thickened coats of the intestine itself. Externally it was very dense in structure; but internally it was soft, and assumed the fungoid, or encephaloid character. In its centre was a vast cavity with irregular parietes, which had furnished the fetid and purulent discharges during life.

It was but very recently that we met with a true fungoid or malignant cancer of the pylorus in a female about 50 years of age. She had experienced daily sickness after food for more than a year; but the complaint was greatly exasperated by long-continued sea-sickness in the steamer between Ostend and London. From that time till the day of her death, six or seven weeks afterwards, she was not able to keep any thing on her stomach. Yet she preserved her corpulency till the last. About three weeks before death we examined carefully the abdomen, and perceived a slight hardness in the region of the pylorus, but it was unattended by tenderness. She was seen by Dr. Granville and Mr. Guthrie in consultation, but no remedial measures could stop the incessant vomiting after even the most trifling quantities of fluid taken into the stomach. The argentum nitratum allayed the irritability of the stomach more than any other remedy; but it gave only temporary relief. The patient was worn out by constant vomiting, fever, and inanition.

On dissection, the parietes of the stomach, in the neighbourhood of the pyloric orifice were thickened and indurated; but from the mucous surface of this thickened part a malignant fungoid tumour projected, and blocked up the passage into the duodenum, which would, with difficulty, admit the point of the little finger. This fungoid prominence was in a state of ulceration. The preparation, which is a remarkably fine specimen of the disease, is in the possession of Mr. Guthrie, at the Eye Infirmary. It is not a little remarkable that the fat was an inch and a half in thickness on the abdomen, notwithstanding this long-continued sickness occasioned by an organic disease of the stomach.

---

### XIX.

### MEATH HOSPITAL, DUBLIN.

[DRS. GRAVES AND STOKES.]

IN the 5th volume of the Dublin Hospital Reports, which we have just received, and of which we shall take ample notice, we find a long and very valuable report from the above hospital, drawn up by Drs. Graves and Stokes. We have selected for this department of our journal, the following cases, reserving the others for subsequent numbers.

